# Development of isoniazid electrochemical sensor using nickel ferrite - nitrogen and sulfur co-doped graphene quantum dot nanocomposite as a new electrode modifier

**DOI:** 10.1038/s41598-024-64797-9

**Published:** 2024-06-20

**Authors:** Mohammad Kazem Ahsani, Fatemeh Ahour, Elnaz Asghari

**Affiliations:** 1 Department of Physical Chemistry, Faculty of Chemistry, University of Tabriz, Tabriz, Iran; 2 Nanotechnology Research Group, Faculty of Chemistry, Urmia University, Urmia, Iran; 3 Department of Nanochemistry, Nanotechnology Research Center, Urmia University, Urmia, Iran

**Keywords:** Electrochemical sensor, N, S doped GQD, Isoniazid, Nickel ferrite, Nanocomposite, Electrocatalyst, Nanoscience and technology, Nanoscale materials, Analytical chemistry, Electrochemistry

## Abstract

This work reports the synthesis of nickel ferrite decorated nitrogen and sulfur co-doped graphene quantum dot (NF@N, S:GQD) and its use as an electrode modifier. The developed NF@N, S:GQD modified glassy carbon electrode (NF@N, S:GQD/GCE) was applied to assess isoniazid (INZ) concentration based on its oxidation at the surface of the proposed electrode. Cyclic voltammetry (CV) and differential pulse voltammetry (DPV) were used as appropriate electrochemical techniques to study the electrochemical behavior of INZ and determine it. Based on combined evidence from surveys, research, and personal results, it is thought that the combination of nickel ferrite and doped graphene quantum dots can synergistically affect results, leading to increased sensitivity and reduced detection limits. This is probably mainly due to the high electrical conductivity of N, S-GQD structure, the electrocatalytic effect of nickel ferrite, and increased surface area resulting from the nano size of the modifier. The optimum conditions for preparing of the modified electrode and determination of INZ are selected by performing electrochemical experiments. The voltammetric response of the sensor is linear from 0.3 to 40 nM INZ under optimal conditions and the detection limit of the sensor is 0.1 nM. The validity and performance of the prepared sensor were confirmed by determining the amount of INZ in the drug and urine as real samples. The composite of doped nanoparticles and nickel ferrite is an innovative modification material to create electrochemical sensors with high sensitivity and selectivity that can be used in pharmaceutical applications.

## Introduction

Isoniazid (INZ) with the chemical formula C_6_H_7_N_3_O, is an antibiotic that is used to treat tuberculosis. Even though INZ has irreversible side effects in long-term use or its overdose, its outstanding biochemical activity has made it an indispensable drug in clinical prescriptions^1–3^. Regular monitoring of patients treated with INZ is necessary to avoid side effects of this drug. For this purpose, a wide variety of methods and sensors have been proposed for the sensitive detection of INZ^4–8^.

The collection of nanoparticles in different sizes, shapes, and compositions has created a revolution in the field of analytical measurement. The use of binary mixed oxides of transition metals is common in electrochemistry and there are reports about their use in electrochemistry^9–16^. In most of these reports, the synergistic effect between two metal oxides is the main reason for their high efficiency. Nickel and iron have always been considered two cheap and environmentally friendly elements in electrochemical applications that form such spinel structures. Nickel ferrite (NiFe_2_O_4_) with an inverted spinel structure as a binary mixed oxide of transition metals shows promising properties as a potential catalyzer^17–19^.

Electrochemical techniques have attracted more attention due to their simplicity, speed of operation, low cost, and so on. The key factor to develop an economic electrochemical sensor with high sensitivity and long-term stability is the design and synthesis of a new electrode modifier^20–22^. Metal oxide nanoparticles (NPs) significantly boost the efficiency of electrochemical sensors due to their catalytic properties. Despite the advantages and wide applications of ferrites, the main problem raised with these mixed oxides, especially in electrochemistry, is their low electrical conductivity^19^. A suitable electrode, in addition to high surface area and electrocatalytic properties, must normally demonstrate a decent level of conductivity, ergo, the use of composites of these oxides along with carbon materials has been practiced commonly in recent years^23–25^.

A new type of carbon-based nanomaterials, called GQDs, has recently attracted much attention due to having high surface area and significant number of edges, which enhance their electrochemical activity and application in sensors. GQDs are usually functionalized or doped with different heteroatoms to enhance intrinsic properties and change electronic structures^26–31^. Doping multiple heteroatoms attracted a lot of attention because it increases the holes on the surface of quantum dots increasing their conductivity and electrochemical activity^32–39^. It is concluded that the problem of low electrical conductivity of ferrites can be solved by composing them with heteroatom-doped GQDs, which makes it possible to use them as electrode modifiers with improved performance and efficiency.

Some electrodes modified with nanomaterials like boron and nitrogen doped mesoporous carbon (BNDC)^40^, zinc and sulfur codoped iron oxide nanocubes anchored on carbon nanotubes^41^, carbon-copper ferrite quantum dots (C-dots@CuFe_2_O_4_)^42^, etc^43–47^, have been used to measure isoniazid. However, the design and synthesis of a new electrode modifier is a key factor in the development of an electrochemical sensor to achieve proper stability and sensitivity.

**Figure 1:**
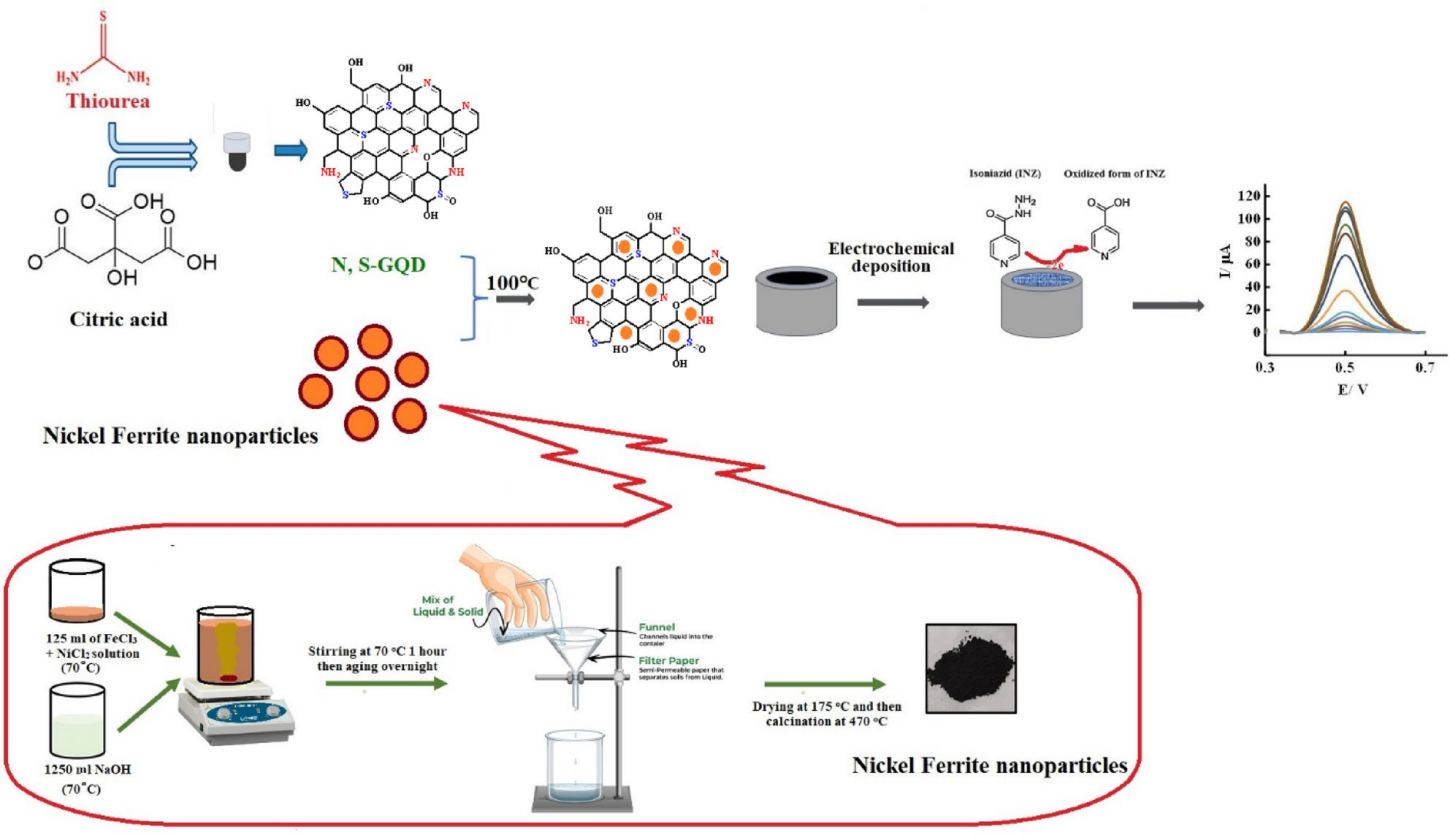
Schematic presentation of prepared sensor.

Considering the mentioned issues and the importance of the green synthesis of new electrocatalysts, for the first time in this research work, we report the successful synthesis and excellent activity of nickel ferrite decorated N, S doped GQD as an electrocatalyst for the measurement of isoniazid (Fig. 1). The presence of N, S:GQD on the electrode surface increases the surface area and electrical conductivity, which, along with the catalytic property of NF nanoparticles, increases the electrochemical signal and decreases the detection limit. The fabricated electrochemical sensor offers numerous advantages, including being cost-effective, highly sensitive, consistently reproducible, and exceptionally selective. Sensitive and selective detection of isoniazid in pharmaceutical samples was performed by the proposed sensor.

## Experimental

### Preparation of nickel ferrite

The nickel ferrite catalyst was prepared by the co-precipitation method. 1.7 g of nickel chloride and 4.225 g of iron (III) chloride were directly dissolved in 125 ml of doubly distilled water and heated to 70 °C. In another vessel, 1.250 L of NaOH solution (0.6 normal) was heated to 70 °C. Then metal precursors were added to the NaOH solution as quickly as possible with vigorous stirring. The obtained solution was forcefully stirred for 1 h at a temperature of 70 °C and kept overnight in the room for aging purposes. The precipitate was filtered and thoroughly washed with hot distilled water to wash away the chloride ions. The separated solid product was dried at 175 °C for 16 h and finally calcined at 470 °C for 3 h to produce a spinel catalyst. The synthesized sample was dried at 175 °C.

### Preparation of NF@N, S:GQD

After synthesizing N, S:GQD as reported in previous work and in Supplementary Information (SI)^39^, 0.04 g of synthesized N, S:GQD in 20.0 mL of water was sonicated for 30 min to form a clear solution. Then slowly and under mechanical stirring, nickel ferrite (0.04 g) was added to the mixture, and the mixture was heated to 100 °C. Unsupported N, S: GQDs were removed by Soxhlet extraction with water for 24 h, and the final product NF@ N, S: GQDs were obtained.

GQDs and NF@GQDs were synthesized similarly without the addition of thiourea.

### Preparation of real samples

Human urine samples were gathered from healthy subjects before the experiment. 50 μl of urine sample was diluted 1:100 with PBS and spiked with different volumes of INZ standard solution before performing the test. To prepare tablet samples, three INZ tablets were finely powdered, mixed, and homogenized. Then a weight equal to one tablet was taken and dissolved in 200 ml of doubly distilled water for 10 min. After centrifugation, the supernatant was diluted to reach the final concentration in the linear range of the calibration curve.

## Results and discussion

### Identification of prepared structures

**Figure 2:**
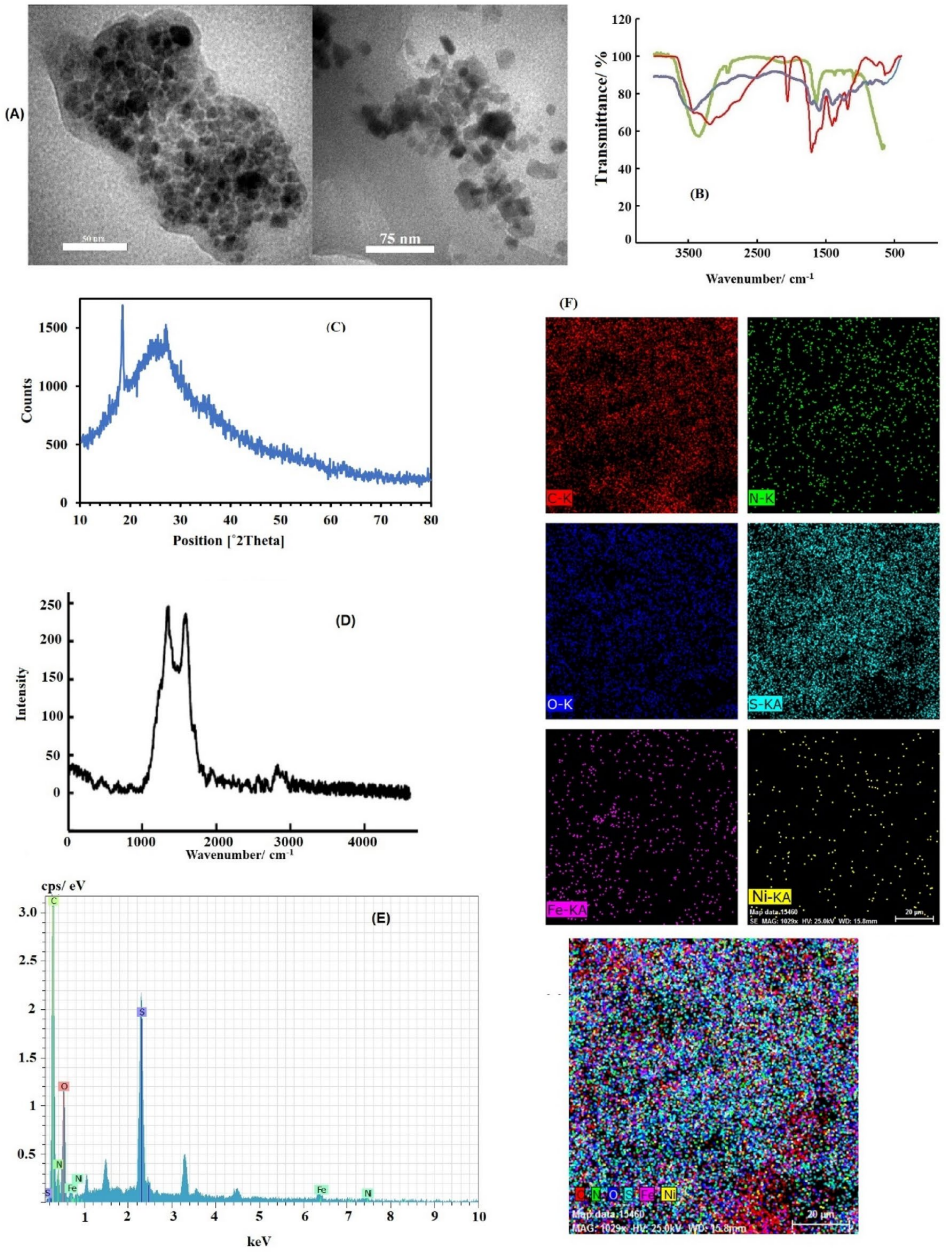
(**A**) TEM image of NF@ N, S:GQD, (**B**) FT-IR diagram for GQD (red), N, S:GQD (green) and NF@N, S:GQD (blue), (**C**) XRD pattern, (**D**) Raman spectra of NF@N, S:GQD, (**E**) EDS spectra of NF@N, S:GQD, (**F**) Elemental mapping of NF@N, S:GQD.

Transmission electron microscope (TEM) imaging was used to determine the structure and size of NF @N, S:GQDs, and the results are shown in Fig. 2A.

According to the presented TEM images, the synthesized nanoparticles have a uniform spherical structure that can be separated from each other.

To investigate the chemical composition and functional groups on the surface of the modifier, infrared spectroscopic analysis (FT-IR) was used, and the related diagram has been shown in Fig. 2B. Broad peaks observed at the obtained spectrum are as follows: corresponding to OH or NH at 3400 cm^−1^, C–H at 2940 cm^−1^, C=O at 1640 cm^−1^, and C=C at 1370 and 1240 cm^−1^. The changes observed at 1090 cm^−1^ are related to N and S doping between carbon atoms of GQD or in other words C–N/C–S bonds, which confirms N, S:GQD synthesis.

In the spectrum of NF@N, S:GQDs, two peaks in the range of 400–600 are observed, which are assigned to the Ni–O and Fe–O bonds in the spinel structure. These results confirm the successful synthesis of NF@N, S:GQDs.

### Examination of X-ray diffraction pattern

X-ray scattering method was used to investigate the structure and crystal areas in different structures of the sample. Figure 2C shows an image of X-ray diffraction related to NF@N, S:GQDs.

Peaks appear where the atoms of a crystal (plane in space) line up. A new type of graphene-like material with greater disorder results in 27° peaks corresponding to (002) planes. The tiny size of GQD leads to the broadening of the XRD peak which prevents the peaks of iron and nickel from being seen in this range.

### Raman investigation

Graphene-based compounds are distinguished by D and G bands in Raman spectroscopy, which are related to the out-of-plane vibrations of C–H bonds that occur in crystal defects. In the present work, D and G bands were appeared at 1345 and 1598 cm^−1^ in the obtained Raman spectrum, which proves the synthesis of N, S:GQD (Fig. 2D).

Also, higher I_D_/I_G_ values result from the presence of N and S in the carbon framework, which is related to the irregular structure and defects created, which improves the electrocatalytic performance of doped nanoparticles.

### Elemental analysis of X-ray energy diffraction diagram

Figure 2E shows the X-ray energy diffraction diagram (EDAX) related to nanocomposite. The peaks related to carbon, oxygen, nitrogen, sulfur, nickel, and iron were appeared in the obtained EDAX spectrum and confirmed nitrogen and sulfur load in GQD and the successful synthesis of NF@N, S:GQD nanocomposite. Also, from the results of elemental mapping, it is clear that almost all points of the surface of the sample are doped with N and S and decorated with nickel and iron. (Fig. 2F).

### Functional evaluations of the prepared sensor

**Figure 3:**
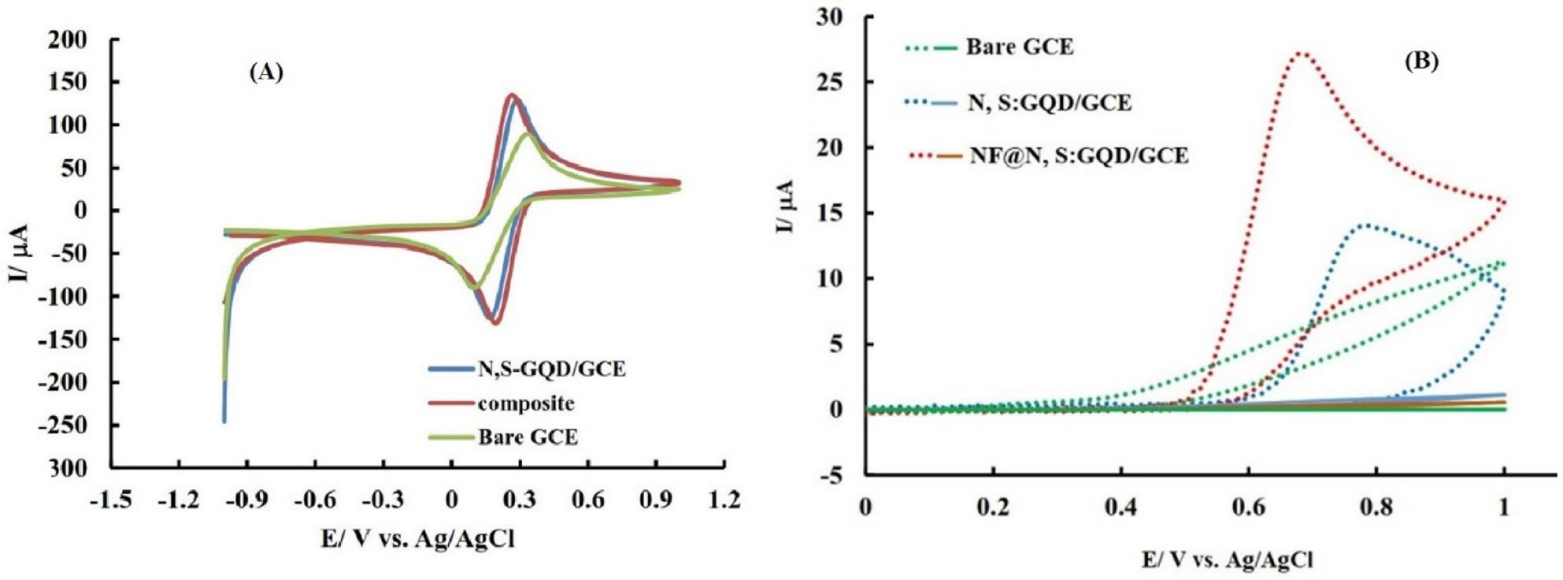
Cyclic voltammograms of the bare and modified electrodes (**A**) after dipping in Fe(CN)_6_^3−^/Fe(CN)_6_^4−^ with a concentration of 5 mM containing 1 M KCl and (**B**) in the blank electrolyte solution before (solid line) and after adding 0.12 mM isoniazid (dotted line). Potential scan speed in (**A**) 100 mV s^−1^ and (B) 50 mV s^−1^.

First and foremost, to explore the immobilization of nanoparticles at the electrode surface and study the efficacy of the nano layer on the results, Fe(CN)_6_^3−^/Fe(CN)_6_^4−^ (ferri/ferro) with a concentration of 5 mM containing 1 M KCl was used as signaling probe. For this purpose, GCE before and after modification with N, S:GQDs or NF@N, S:GQDs was immersed in ferri/ferro, and its cyclic voltammetry was done in the potential range from 0 to 1 V (Fig. 3A).

Based on the obtained results, the modified electrodes have a higher electrical conductivity (EC) and a larger electrochemical active surface (EAS), which shows itself as a decrease in the anode–cathode peak separation and an increase in the peak current. The EAS of the NF@N, S:GQD/GCE was calculated as 0.17 using the Randles–Sevcik equation based on obtained voltammograms in a 5 mM Fe(CN)_6_^3−^/Fe(CN)_6_^4−^ solution containing 1 M KCl with varying scan rates^48^. The obtained EAS of the modified electrode is greater than geometric surface area of GCE which is equal to 0.031.

Also, the recorded voltammograms confirm the better performance of NF@N, S:GQD than N, S:GQD based on lower peak separation and higher peak current in comparison. Considering that EAS and EC are effective factors in the amount of current, it can be claimed that the stabilization of NF@N, S:GQD at the GCE has a positive effect on the electrochemical performance of the prepared sensor.

Isoniazid oxidation in many cases requires a high overpotential and produces a low current intensity. To check the efficiency of the modified electrodes for the measurement of isoniazid, the cyclic voltammetric signal of the bare and modified GCE inside the background electrolyte was recorded before and after the addition of 0.12 mM isoniazid, and the results are shown in Fig. 3B.

The intensity of the resulting peak at the surface of the NF@N, S:GQD/GCE is higher and sharper than that obtained at the bare and N, S:GQD/GCE and appeared at low potentials. Based on these results, it can be said that the NF@N, S:GQD modified electrode shows a suitable electrochemical behavior for INZ oxidation. Also, comparing the results obtained in the case of INZ and ferri/ferro confirms the electrocatalytic effect of NF for the oxidation of INZ, which manifests itself in the appearance of a greater difference in the INZ oxidation signal.

### Optimizing electrode modification conditions

Considering that, some experimental variables such as the modifier immobilization method, and the amount of modifier are effective factors in the electrode^’^s stability and performance, experiments were arranged to select and optimize the experimental variables as presented below.

### Modification method

One of the effective factors in making a good sensor is the suitable amount of fixed modifier on the electrode surface. For this purpose, the effect of electrode modification method such as casting and electrochemical method was investigated based on the INZ signal.

The obtained results (Fig. S1) show an increase in the analyte signal at the surface of the electrochemically modified electrode and its more stability compared to the other one. The stability of the modified electrode was evaluated by performing repeated potential cycling in the INZ solution. The results showed that the electrochemically modified electrode compared to the casting modified electrode, which is stable up to 8 cycles, has unchanged results (variation lower than 5%) up to 37 cycles. Therefore, NF@N, S:GQD was immobilized using the electrochemical method at the GCE surface.

### Optimizing the number of potential scans to fix the modifier

Since the insufficient modification of the electrode with composite cannot create enough active sites on the electrode surface, the effect of operational factors on the obtained results was studied. Among the important and effective factors in the electrochemical stabilization method are the number of cycles, the potential scan speed and the potential scan range, all of which were studied and after conducting the relevant tests, the optimal conditions for the preparation of the electrode were selected.

It could be said that by applying 60 cycles, enough amount of NF@N, S:GQD stabilize at the surface and increasing the number of cycles up to higher values does not affect effect on the resulting signal. Therefore, to save time, 60 cycles were chosen as the optimal value for stabilizing the modifier (Fig. S2).

Experiments related to the effect of the potential scan range were also considered and the electrode modified by potential scanning from 0 to 1 V (data not shown) showed the best results which electrostatic interactions between the modifier and the electrode surface may be the cause of it.

The effect of the potential scanning speed on the isoniazid oxidation signal was also investigated and based on the results, at scanning speeds from 25 to 100 mV/s the same signals were obtained, which indicates sufficient stabilization of the modifier at the electrode surface, but with increasing the potential scanning speed to 150 mV/s, isoniazid oxidation signal decreased. Probably, as the potential scan rate increases to 150 mV/s and above, there is not enough time for the composite to stabilize on the surface, leading to a reduction of the active sites and the corresponding oxidation signal.

Therefore, to reduce the time required to modify the electrode (saving time), 100 mV/s was chosen as the optimal scan rate for the electrodeposition of the modifier (Fig. S3).

After optimizing the electrode preparation conditions, the prepared electrode was used in optimal conditions for the measurement of the isoniazid drug, and the effective parameters in the measurement of INZ were selected.

### Optimizing the effective factors

Studies show that the background electrolyte can be as effective as the amount of the modifier in the detection process. In this way, the effect of factors such as the type, concentration, and pH of the electrolyte were also investigated in the results.

### pH effect

**Figure 4:**
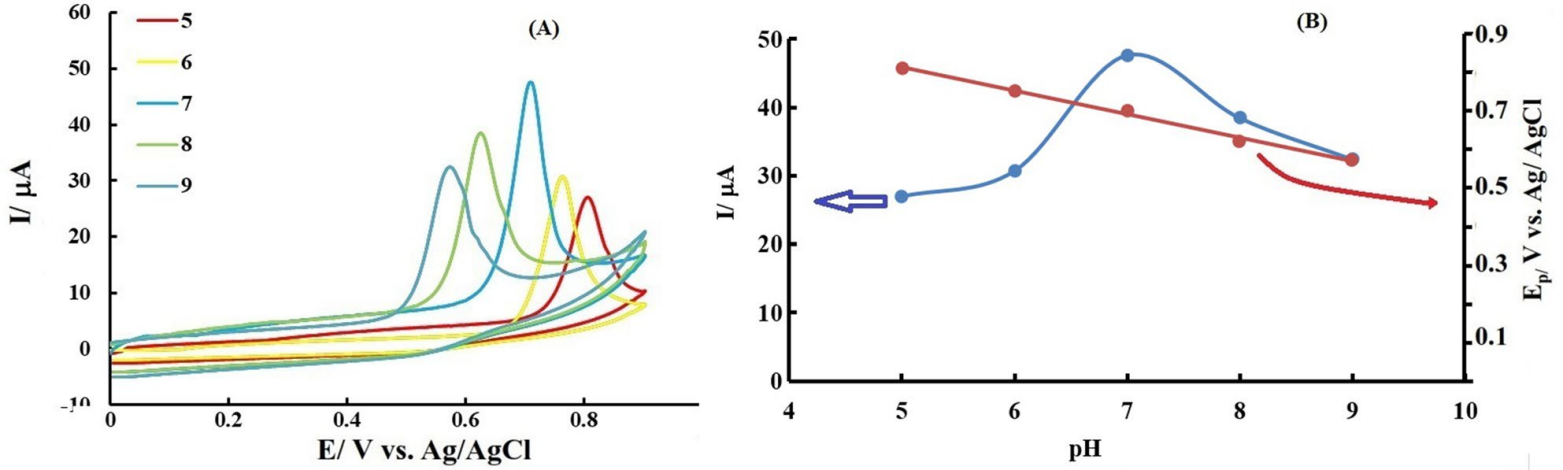
(**A**) Cyclic voltammograms of the modified electrode after dipping in PBS containing 0.25 mM INZ with different pH values: 5, 6, 7, 8 and 9, (**B**) variation of (a) INZ electrooxidation peak current and (b) INZ electrooxidation peak potential versus pH. Potential scan rate: 50 mV/s.

The voltammograms obtained using modified electrode in solutions with different pH values are shown in Fig. 4A.

As can be seen with decreasing pH, the oxidation peak of isoniazid analyte shifts towards positive potentials and its current intensity (peak height) decreases. The graph of the peak current variations versus pH is shown in Fig. 4B. Based on the results, the maximum current is observed at a neutral pH; therefore, a pH equal to 7 was chosen as the optimal pH value.

**Figure 5:**
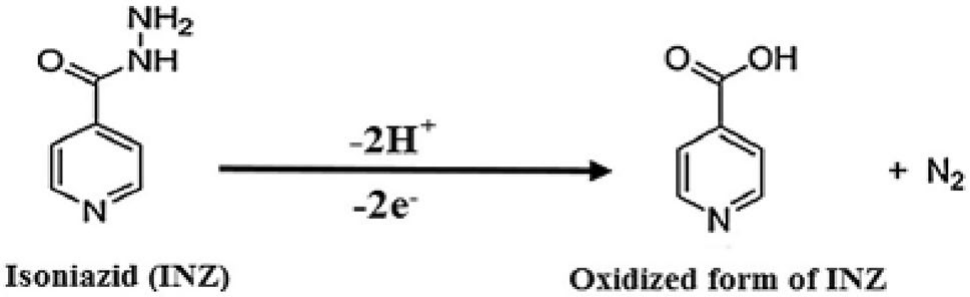
The proposed oxidation mechanism of INZ.

In Fig. 4B, the graph of the variation in anodic peak potential, E_pa_, is shown in terms of pH, which has a slope equal to 61 mV/pH. The obtained slope of 61 mV is close to the theoretical value of the Nernst slope of 59 mV, which shows that in the electrooxidation of INZ at the proposed sensor, an equal number of electrons and protons are involved, which agrees with the mechanism proposed in the articles based on the exchange of two protons and two electrons. Based on this observation and the proposed mechanisms for the oxidation of isoniazid in articles^44^, the following mechanism for the oxidation of INZ at the surface of the proposed sensor can be suggested (Fig. 5):

### The effect of the background electrolyte

The type of buffer is considered an effective factor for performing the detection process, so considering the effect of pH on the results and the optimal pH equal to 7, the effect of two background electrolytes including 0.1 M phosphate buffer and 0.1 M tris–HCl both containing 0.5 M potassium nitrate were studied. Based on the observations (Fig. S4), the results were similar in both buffers and due to the availability of phosphate buffer, this was chosen as the optimal buffer.

### Effect of electrolyte concentration

By using different concentrations of phosphate buffer and potassium nitrate, the effect of background electrolyte concentration on the voltammetric signal of INZ was investigated.

Based on the results, the signal obtained in concentrations greater than 0.5 M potassium nitrate in the absence and presence of phosphate buffer is equal and has no difference (not shown). In other words, by using this concentration, there is enough conductivity in the solution to transfer charge. According to the proposed mechanism and optimal pH  7, to ensure pH stabilization, PBS (0.1 M phosphate buffer and 0.5 M potassium nitrate) was selected as the background electrolyte. Also, cyclic voltammograms of NF@N, S:GQD/GCE in PBS containing different concentrations of isoniazid showed a linear relationship between current and INZ concentration (Figures S5, S6).

### The effect of potential scanning rate on the isoniazid oxidation process

**Figure 6:**
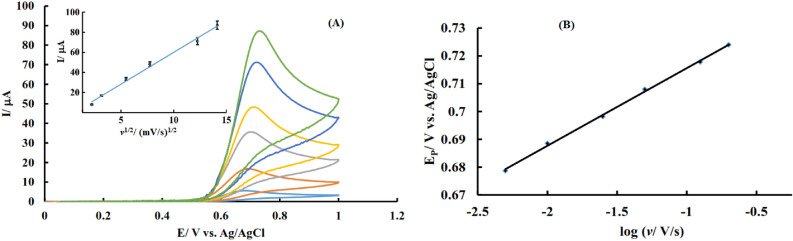
**(A)** Cyclic voltammograms of the modified NF@N, S:GQD/GCE in 0.25 mM INZ solution with different potential scanning speeds; inset of (**A**): variation of INZ electrooxidation peak current according to the square root of potential scan rate; **(B)** variation of INZ electrooxidation peak potential versus logarithm of the potential scan rate.

According to the available reports, in a diffusion-controlled process, the peak current of the electroactive species is proportional to the square root of the potential sweep rate, and for surface adsorbed species, it is proportional to the potential sweep rate. In order to understand the kinetics of electrochemical oxidation of INZ at NF@N, S:GQD/GCE, the effect of the potential scanning speed on the resulting voltammetric signal was studied by recording voltammograms using different potential scan rates in 0.3 mM INZ solution, and the results are shown in Fig. 6.

The graph of peak current changes according to the square root of the potential sweep rate is shown in the inset of Fig. 6A. According to the obtained results, the anodic peak current increases proportionally to the square root of the potential scan rate, which indicates that the electrochemical oxidation of INZ on the surface of this electrode is a diffusion-controlled process.

One of the electrochemical ways to evaluate the number of electrons involved in the rate-determining step of the reaction is to examine the dependence of the peak potential on the speed of the potential scan rate. For a completely irreversible process controlled by diffusion and in the absence of any kind of ohmic drop, the relationship between the peak potential and the logarithm of the potential sweep rate (log *v*) is expressed by the equation:

$${\text{E}}_{\rm p} = \left( {{\text{b}}/2} \right) \, \log v + {\text{ Const}}$$

where E_p_, *v*, and b are related to the peak potential, potential scan rate, and Tafel slope, respectively.

Figure 6B shows the variation of INZ electrooxidation peak potential versus log *v* using that curve the Tafel slope obtained is equal to 56 mV/decade.

By inserting the obtained Tafel value in the equation 2.3RT/(1 – α)n_α_F, the value of α and the number of electrons participating in the rate determining step, n_α_, were calculated 0.48 and 2 respectively.

### Quantitative detection of isoniazid

**Figure 7:**
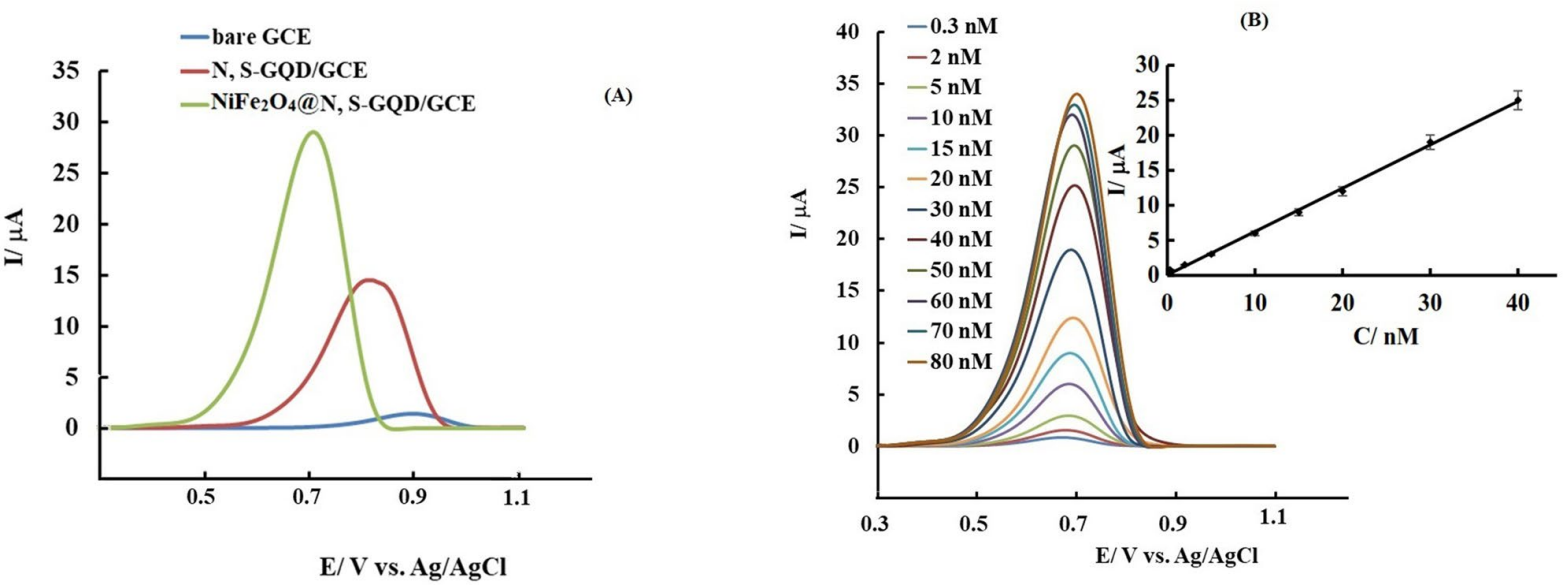
(**A**) DPV results of bare and modified electrodes in PBS after addition 40 nM of INZ; (**B**) DPV results of the modified electrode inside PBS after adding different concentrations of isoniazid; inset (**B**): variation of peak current versus concentration related to the presented voltammograms.

The electrochemical activity of the modified and unmodified electrodes against INZ was investigated to increase the sensitivity and reduce the detection limit of the method. Figure 7A presents the differential pulse voltammograms of the unmodified and modified GCE after placing it inside the phosphate buffer solution containing 40 nM INZ.

As can be seen in the resulting voltammograms, a very small peak is observed on the surface of the bare electrode, and its height increases after modification of the electrode, which can be related to the effect of nanoparticles placed at the surface. Also, the resulting peak at NF@N, S:GQD modified GCE is larger than that modified by N, S:GQD, which is related to the catalytic effect and synergistic consequence between doped GQD and NF nanoparticles, which increases the rate of charge transfer and electrocatalysis of isoniazid oxidation. In simpler terms, the modified electrode with NF@N, S:GQD showed a much better and sharper peak in cyclic voltammetry, which is consistent with the present results.

### Investigating the effect of pretreatment parameters

Based on the previous reports for INZ measurement, in some cases, the absorption of this compound has been observed at the electrode surface. Therefore, in this work, the effect of pre-concentration time and pre-concentration potential was investigated. For this purpose, after preparing the modified electrode and floating it along with the auxiliary and reference electrodes inside the carrier electrolyte, 40 nM of isoniazid was added into the solution, and the corresponding differential pulse voltammogram in four states (1: immediately, 2: after 5 min immersion without applying potential, 3: after 5 min immersion with applying 0.3 V or −0.3 V as accumulation potential) were recorded (Fig. S7).

The obtained results showed that INZ could not be adsorbed on the modified electrode surface, which was consistent with the cyclic voltammetry results.

### Examining the effect of INZ concentration

For this purpose, the modified electrode was floated in the PBS and the potential scan was performed in the optimal conditions obtained from the previous parts using the DPV method. Then certain amounts of INZ solution with a known concentration were added to the electrochemical cell and the corresponding voltammograms were recorded after each increase. The obtained voltammograms are shown in Fig. 7B.

### Determining the limit of detection (LOD)

One of the parameters that differentiates an analytical method for measuring a sample is the LOD of the method. LOD is the concentration of the analyte that has a response equal to the response of the blank sample plus three times of the standard deviation of the blank sample (S):

$${\text{y}}_{{{\text{LOD}}}} = {\text{ y}}_{{\text{b}}} + {\text{ 3S}}_{{\text{b}}}$$

In this equation, S_b_ represents the standard deviation of the blank solution or the standard deviation of the calibration curve. According to the standard deviation of the blank sample and the slope of the calibration curve, the LOD of the proposed sensor was calculated as 0.1 nM.

For the comparison of the performance of the proposed sensor with the previous ones, the results obtained using this sensor are compared with the methods presented in the articles and summarized in Table 1.

**Table 1:** Comparison analytical parameters of NF@N, S:GQD/GCE with other modified electrodes as INZ sensor.

Modified electrode	Detection limit	Linear range	Ref
BNDC	1.5 nM	0.02–1783 μM	^40^
C-dots@CuFe_2_O_4_	0.041 μM	0.1–14.0 μM	^42^
Ag–P(MMA-co-AMPS)	10 nM	50.0 nM–150.0 μM	^43^
V_2_O_3_-C@PB/GF	0.83 nM	2.5 nM–1.1 mM	^44^
FeCoSe_2_/GCE	0.124 nM	0.03–1.0 μM	^45^
BiO-SPEs	1.85 µM	5–1760 µM	^47^
NF@N, S:GQD/GCE	0.1 nM	0.3–40 nM	This work

*BNDC* B/N co-doped mesoporous carbon, *Ag–P*(*MMA-co-AMPS*) methyl methacrylate and 2-acrylamido-2-methylpropane sulfonic acid (P(MMA-co-AMPS)) and silver nanoparticles (Ag NP), *V*_*2*_*O*_*3*_*-C@PB/GF* carbon doped vanadium trioxide @ Prussian blue supported on graphite felt, *FeCoSe*_*2*_ bimetallic cobalt-iron diselenide, *BiO-SPEs* bismuth oxide (Bi_2_O_2.33_), nanostructures (nanorods).

The obtained results prove that the proposed sensor in this research has good sensitivity and reproducibility, and due to its low cost, easy preparation, and simple use, it is a valuable analytical work.

### Investigation of the effect of possible interferences

Investigating the negative effects of the presence of other drugs such as acetaminophen (AC), rifampin (RIF), vitamin C (AA), uric acid (UA) and folic acid (FA) in measuring INZ by the proposed modified electrode was done. For this reason, firstly, the voltammetric signal was recorded in the presence of 10 nM INZ, and then again after the addition of increasing amounts of probable interfering drugs to the analyte solution. The obtained voltammetric signals were compared with the initial voltammetric signal. If the difference between these two signals is less than 5%, it shows that INZ can be measured with proper accuracy in the presence of these species. The results of this investigation showed that the mentioned compounds have no interference with the obtained results until tenfold concentration (Fig. S8). This may be due to the different electrooxidation potential of these compounds or the lower sensitivity of the proposed sensor to these drugs. Therefore, it can be said that these analytes do not interfere with the measurement of isoniazid.

### Measurement of isoniazid in real samples

The modified electrode was used as an electrochemical sensor for the measurement of INZ in pharmaceutical samples using the standard addition technique.

For performing the standard addition method, the electrode was placed in 10 ml of analyte solution (PBS containing a certain amount of drug or spiked urine) as a blank, and the corresponding voltammograms were recorded before and after adding certain amounts of the isoniazid standard sample. The recorded currents were drawn according to the added standard concentration and after extrapolation, the concentration of the real sample was determined. The values ​​obtained from the voltammetry method are consistent with the values ​​mentioned on the drug or added to the urine, which shows the applicability of the proposed sensor for measuring isoniazid in real samples.

The results presented in Table 2 confirm that the proposed sensor could analyze real samples without significant errors.

**Table 2:** Results were gotten for the measurement of INZ in real samples.

Sample	INZ added (nM)	INZ found (nM)	Recovery (%)	RSD (%)^a^
INZ tablet	0	9.6		1.9
	5	14.84	101.6	2.5
	10	19.3	98.5	3.8
	15	24	97.6	3.2
Urine	0	0		
	5	5.04	100.8	3.9
	15	14.8	98.7	3.1

^a^Relative standard deviation values based on three repetitions.

### Examination of electrode surface repeatability and reproducibility

Electrode surface repeatability is important for performing reliable and fast tests. The results of numerous tests (Fig. S9) showed that as isoniazid adsorption does not take place at the electrode surface, no treatment is necessary to perform multiple tests with a prepared electrode, and the electrode can be reused up to 15 times only by simple washing with distilled water.

By measuring the same concentration of the analyte using 5 modified electrodes under the same conditions, the reproducibility of this sensor was assessed, and the results showed that the signal with an acceptable standard deviation is obtained at the surface of all electrodes, which shows the reproducibility of the sensors prepared in this research.

The long-term stability of the modified electrode was also discussed. For this purpose, the response of the freshly prepared electrode immersed in 5 nm INZ was compared with the results obtained after different times. The histogram related to the signal changes after different times is shown in Fig. S10. The results show that the proposed electrode has good stability and can be used up to 13 days after preparation.

## Conclusions

It looks like you're referring to the successful production and application of nickel ferrite decorated N, S doped GQD as an electrode modifier for the construction of an electrochemical isoniazid (INZ) sensor. The accurate and precise determination of INZ concentration was achieved by using the DPV method in both drug and urine samples with the newly developed NF@N, S:GQD modified GCE. The combination of nickel ferrite and doped graphene quantum dots appears to have a synergistic effect on the results, leading to an increase in sensitivity and a decrease in the detection limit. The high electrical conductivity of the N, S:GQD structure, the electrocatalytic effect of nickel ferrite, and the increased surface area from the nano size of the modifier primarily contributed to the results. The sensor demonstrated excellent ability for the sensitive and selective determination of INZ and its applicability for the determination of INZ in spiked human urine and drug tablets.

### Supplementary Information

Supplementary Information.

## Data Availability

All data generated or analysed during this study are included in this published article and its supplementary information files.
