# Supplementary material for: Development of isoniazid electrochemical sensor using nickel ferrite - nitrogen and sulfur co-doped graphene quantum dot nanocomposite as a new electrode modifier
Source: Sci Rep. 2024 Jun 20;14:14228. doi: 10.1038/s41598-024-64797-9 (PMC11189936; doi:10.1038/s41598-024-64797-9)
Supplement: Supplementary file 1 — Supplementary Information. [file 41598_2024_64797_MOESM1_ESM.docx]

**Development of isoniazid electrochemical sensor using nickel ferrite - nitrogen and sulfur co-doped graphene quantum dot nanocomposite as a new electrode modifier**

M. K. Ahsani^a^, F. Ahour*^b, c^, E. Asghari*^a^

^a^ Department of Physical chemistry, Faculty of Chemistry, Tabriz University, Tabriz, Iran

^b^ Department of Nanochemistry, Faculty of Chemistry, Urmia University, Urmia, Iran

^c^ Nanotechnology Research Center, Urmia University, Urmia, Iran

Corresponding author:

* Fatemeh Ahour

E-mail: [Fatemeh.ahour@gmail.com](mailto:Fatemeh.ahour@gmail.com), f.ahour@urmia.ac.ir; Fax: +98 44-32752746

Experimental

Instrumentation and reagents

To perform electrochemical tests, the AUTOLAB PGSTAT 30 electrochemical analysis system was used, which is connected to the controlling computer through the GPES 4.9 software package. A three-electrode set consisting of PdNPs@N-GQD modified glassy carbon electrode (diameter 3 mm) as working electrode, Ag/AgCl (1 M KCl) and platinum wire as a reference, and auxiliary electrodes respectively were used in experiments.

A Nicolet FT-IR NEXUS 670 spectrometer (Thermo Scientific, USA) was used to record IR spectra and identify different functional groups in the synthesized compounds. MIRA III scanning electron microscope connected to EDX-Line scan map was used to perform field effect scanning electron microscope (FE-SEM) and energy dispersive X-ray spectroscopy (EDX). The pH value was measured using a digital pH meter (HANNA 212). The GCE surface was cleaned using an ultrasonic bath (KODO model JAC1002). This device was also used to prepare the homogenous solution of the modifier. All chemicals with analytical grade were obtained from Merck, Germany. Deionized water was used to prepare all solutions. INZ solutions were prepared freshly just before experiments.

Voltammetric measurements

For CV (cyclic voltammetry) measurements, INZ was added in electrolyte containing electrochemical cell and analysed using potential scanning in the range from 0 to 1 V. In DPV analysis, potential was scanned from 0 to 1.2 V with the best DPV parameters as 0.4 s interval time, 0.05 s modulation time, 25 mV potential amplitude, and 5 mV step potential. All the experiments were done at least 3 times and the presented results are the average of these replicates with the corresponding error bars in the figures.

**Preparation of N, S:GQD**

For the synthesis of N, S:GQD, 0.21 g of citric acid (1 mmol) and 0.23 g of thiourea (3 mmol) were dissolved in 5 ml of deionized water and stirred until a clear solution was obtained. Then the resulting solution was heated in an autoclave at 160 ˚C for 4 h. Synthesized nanoparticles were collected by adding ethanol and separated by centrifugation at 5000 rpm for 10 minutes. The resultant solid can be dispersed in water and used to modify the electrode.

**Electrode modification**

A suspension of NF@N, S:GQD was prepared by dispersing 10 mg modifier in 10 ml of phosphate buffer (1 mg/ml ) and placed in an ultrasonic bath for 1 h to become a homogenous solution. This suspension can be used for one month. Each time before use, this suspension was placed in an ultrasonic bath for 15 minutes and exploited for the preparation of the working electrode.

**Results and Discussion**


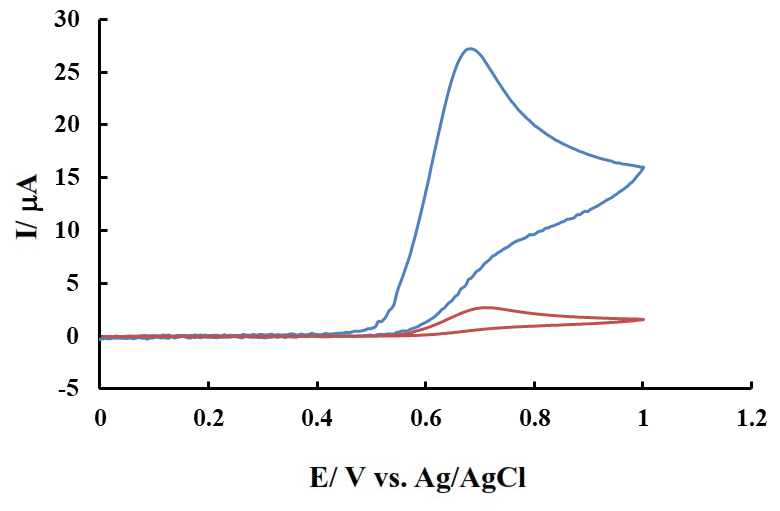


**Fig. S1.** Investigating the immobilization method of NiFe_2_O_4_@N, S:GQD at the surface of GCE relying voltammetric signal of 0.12 mM INZ (red diagram: droplet, blue diagram: electrochemical)


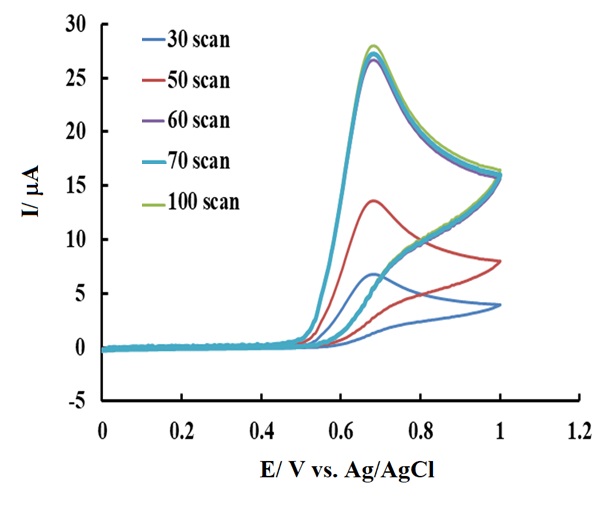


**Fig. S2.** Cyclic voltammograms of the NF@N, S:GQD modified GCE prepared using different number of cycles after dipping in PBS pH 7 containing 0.12 mM INZ; Scan rate: 50 mV s^-1^.


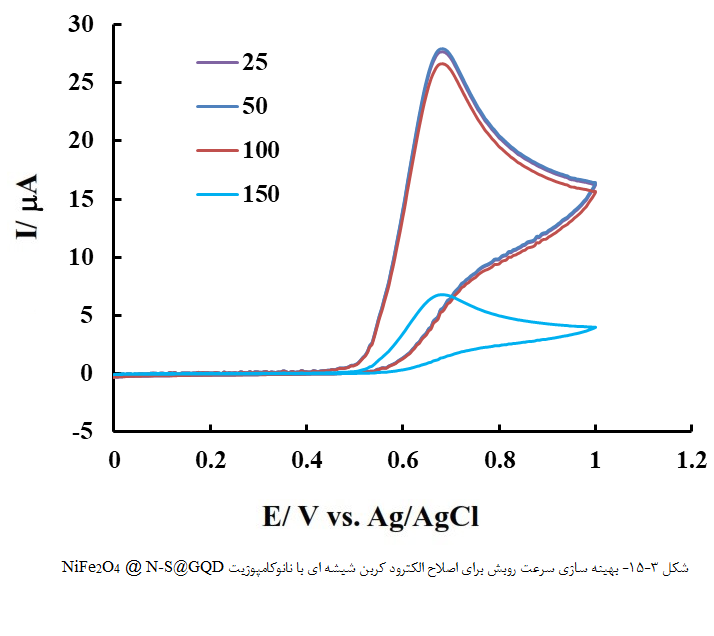


**Fig. S3.** Cyclic voltammograms of the NF@N, S:GQD/GCE prepared by electrochemical method using different scan rates after dipping in in PBS pH 7 containing 0.12 mM INZ; Scan rate: 50 mV s^-1^.


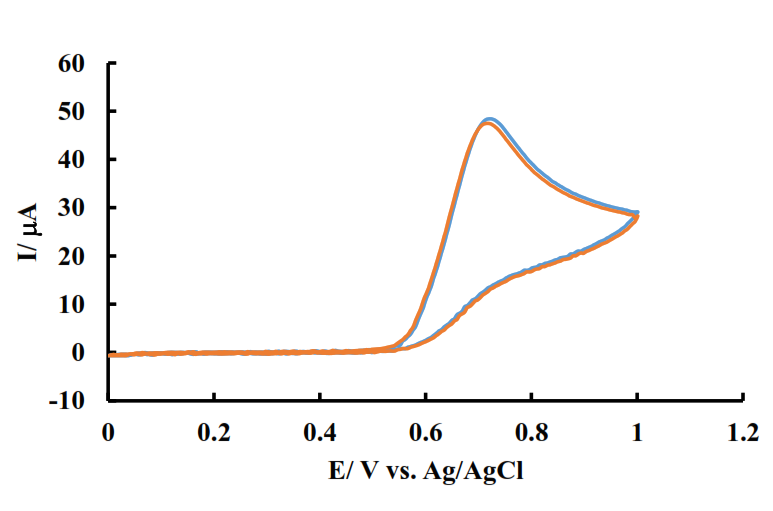


**Fig. S4.** Cyclic voltammograms of the NF@N, S:GQD/GCE after dipping in 0.1 M of: (red curve) tris-HCl and (blue curve) PBS with pH 7 containing 0.25 mM INZ; Scan rate: 50 mV s^-1^.


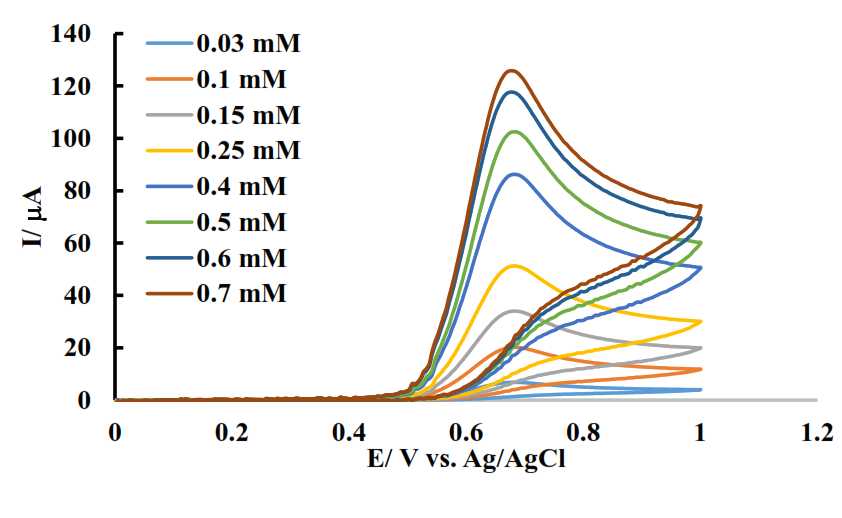
**Fig. S5.** Cyclic voltammograms of NF@N, D:GQD/GCE in the presence of different concentrations of INZ.


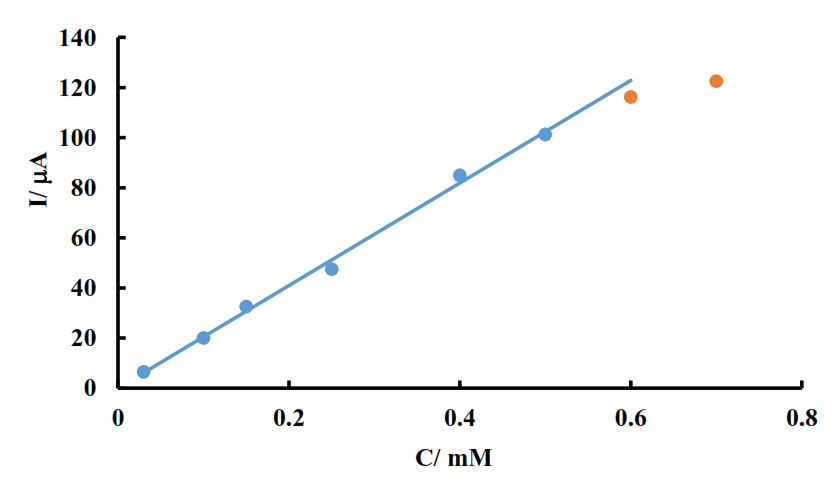


**Fig. S6.** Variation of anodic peak current versus isoniazid concentration; Scan rate: 50 mV s^-1^.


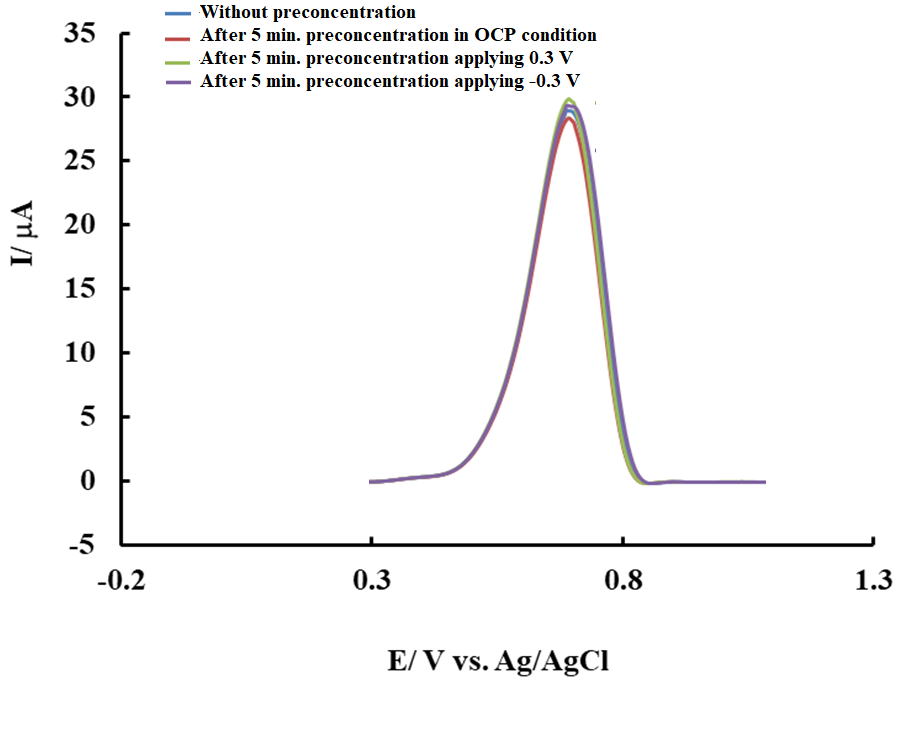


**Fig. S7.** Differential pulse voltammograms of modified electrode recorded in PBS after addition 40 nM INZ applying different preconcentration conditions.


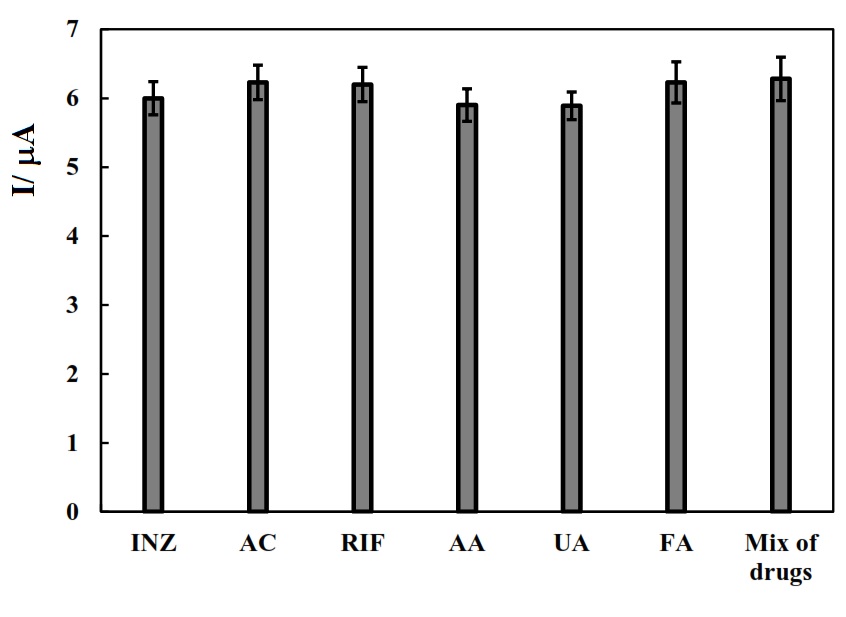


**Fig. S8.** Histogram related to DPV response of NF@N, S:GQD/GCE dipped in PBS with pH 7 after addition of 10 nM of INZ and other drugs with concentration of 100 nM as interfering species and a mixture of these drugs.


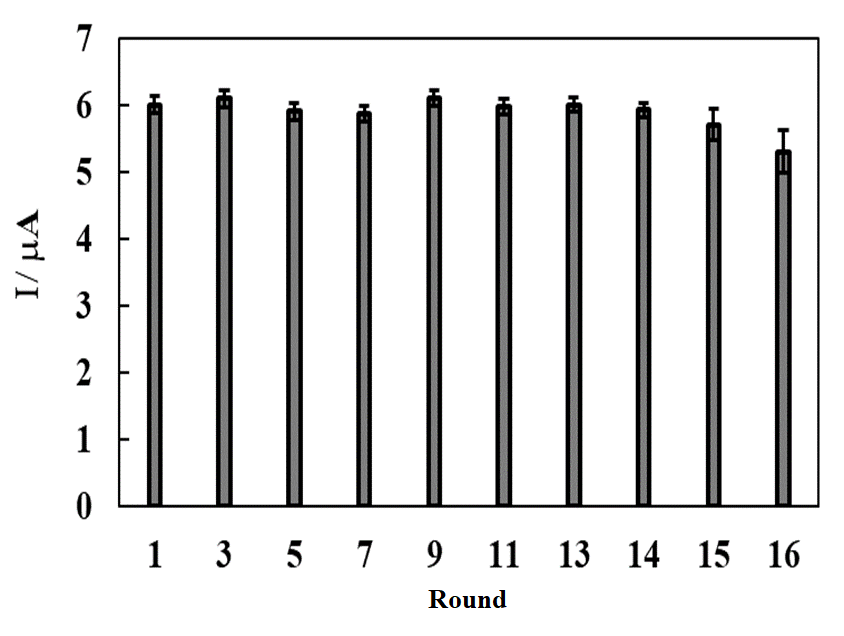


**Fig. S9.** Histogram related to DPV response of NF@N, S:GQD/GCE dipped in PBS with pH 7 containing 10 nM INZ in different repetitions .


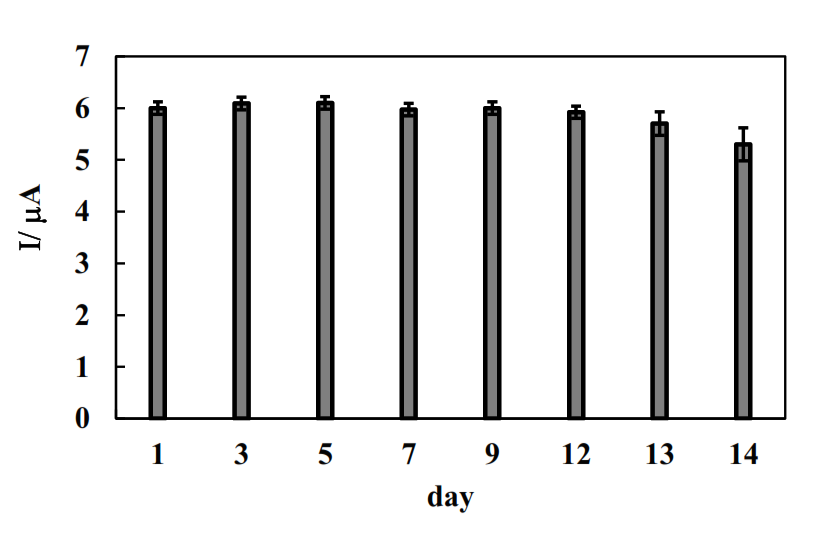


**Fig. S10.** Histogram related to variation of DPV signal of NF@N, S:GQD/GCE after immersion in 10 nM of INZ using newly prepared electrode and after storage for different days
